# The HSN egg-laying command neurons are required for normal defecation frequency in *Caenorhabditis elegans* (II)

**DOI:** 10.17912/10.17912/micropub.biology.000094

**Published:** 2019-03-29

**Authors:** Jessica Garcia, Kevin M. Collins

**Affiliations:** 1 Department of Biology, University of Miami, Coral Gables, FL 33146

**Figure 1:**
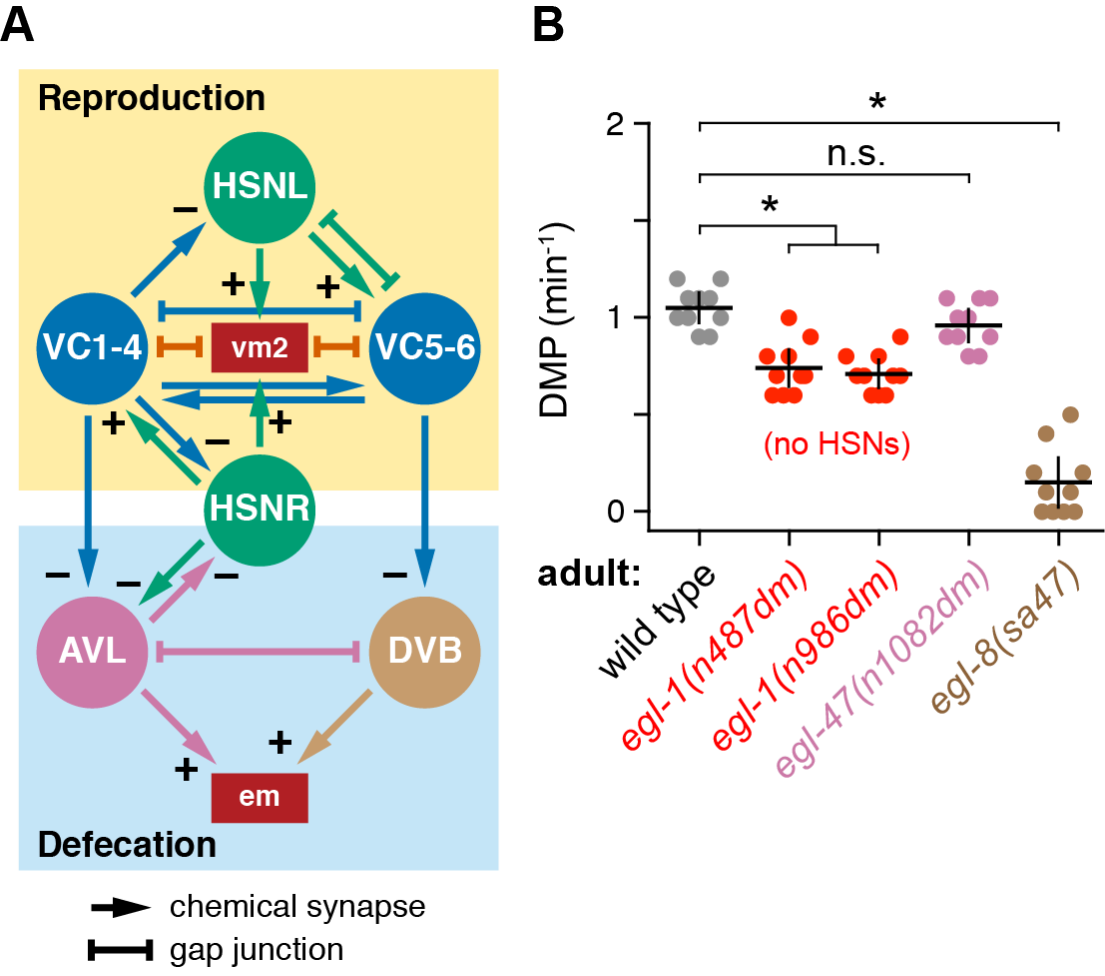
(**A**) Wiring diagrams of the reproductive circuit (top) and defecation motor circuit (bottom). HSN (green) and VC (blue) neurons synapse onto each other and the vm2 muscles for egg laying. Data from White J.G. et al. (1986) indicate HSN and VC also make and receive synapses from AVL and DVB, excitatory GABA motor neurons that regulate the contraction of the enteric muscles (em) for defecation. Arrows indicate chemical synapses, and + or – indicates a presumptive excitatory or inhibitory synapse, respectively. Bar-headed lines indicate gap junctions (e.g. electrical synapses). (**B**) Scatterplots showing average DMP frequencies (min^-1^) from ten wild-type (grey), HSN-deficient *egl-1(n487dm)* and *egl-1(n986dm)* mutants (red), gain-of-function *egl-47(n1082dm)* (pink), and *egl-8(sa47)* PLCβ null mutant adults (brown) after recording for 5 min. Error bars indicate the 95% confidence interval for the mean. Asterisk indicates p<0.0001; n.s. indicates p=0.5208 (One-way ANOVA with Bonferroni’s correction for multiple comparisons).

## Description

In our accompanying paper, we show that Ca^2+^ activity in the HSN egg-laying command motor neurons is associated with a delay in the frequency defecation motor program (DMP) in both late L4 juveniles and egg-laying adults (Ravi and Collins 2019). While optogenetic activation of the HSNs did not directly reduce the frequency of defecation, egg-laying adults did show a significant reduction in defecation frequency compared to younger animals, consistent with previous results (Bolanowski *et al.* 1981). As shown in Figure 1A, the egg-laying and defecation circuits are interconnected. The HSN command neurons and VC motor neurons that synapse onto the egg-laying vulval muscles also make and receive synapses from the excitatory GABAergic AVL and DVB motoneurons that innervate the enteric muscles that regulate defecation. We test the functional relationship between egg-laying and defecation behaviors, we measured DMP frequency in animals with altered HSN neurotransmitter signaling. We hypothesized that mutations that reduce HSN neurotransmitter signaling would *increase* DMP frequency because periods of elevated HSN Ca^2+^ activity were associated with decreased DMP frequency (Ravi and Collins 2019). Surprisingly, animals bearing two independent *egl-1(dm)* mutants that cause the HSNs to undergo premature cell death showed a *decrease* in DMP frequency (Figure 1B). While this delay was significant, it was mild compared to *egl-8(sa47)* null mutants that eliminate PLCβ and IP_3_ signaling required for proper timing of the defecation motor program (Dal Santo *et al.* 1999; Lackner *et al.* 1999). These results show the HSNs are required for a normal DMP rhythm in adult animals. Interestingly, this delay in defecation frequency in HSN-deficient animals was not observed in *egl-47(dm)* mutant animals with strong defects in HSN neurotransmitter release and similar defects in egg-laying behavior as *egl-1(dm)* mutants (Moresco and Koelle 2004). We do not believe this *egl-1(dm)*-specific defect in defecation is caused by differences in bloating in response to egg accumulation in the uterus, as both mutants retain an indistinguishable number of embryos (Moresco and Koelle 2004) and show similar delays in the onset of egg laying (Ravi *et al.* 2018). Further, *egl-47(dm)* animals still have HSNs (Moresco and Koelle 2004) and show occasional HSN Ca^2+^ transients, so the reduced DMP frequency in *egl-1(dm)* animals suggests the HSNs are developmentally required for a normal DMP rhythm and/or that even low levels of serotonin or NLP-3 neuropeptide release from HSN are sufficient for a normal defecation rhythm (Brewer *et al.* 2019). We propose that animals coordinate egg-laying and defecation behaviors to direct changes in hydrostatic pressure used to drive expulsion of uterine or intestinal contents, but that HSN signaling is still required for full coordination of these behaviors. Consistent with this hypothesis, a common set of signaling molecules regulate activity of circuits that modulate egg-laying and defecation behaviors (Reiner *et al.* 1995).

## Reagents

Strains available from CGC: Bristol N2, MT1082 *egl-1(n487dm)* V; MT2248 *egl-47(n1081dm)* V; and JT47 *egl-8(sa47)* V. Strain available upon request: MIA26 *egl-1(n986dm)* V. DMP frequency was measured based on the timing of the final expulsion step in animals staged 24 hours after the late L4 stage (Liu and Thomas 1994).
